# Investigating the role of *Osiris *genes in *Drosophila sechellia *larval resistance to a host plant toxin

**DOI:** 10.1002/ece3.4885

**Published:** 2019-01-15

**Authors:** Stephen M. Lanno, Serena J. Shimshak, Rubye D. Peyser, Samuel C. Linde, Joseph D. Coolon

**Affiliations:** ^1^ Department of Biology Wesleyan University Middletown Connecticut

**Keywords:** ecological genetics, host specialization, octanoic acid

## Abstract

The underlying genetic basis of adaptive phenotypic changes is generally poorly understood, yet a growing number of case studies are beginning to shed light on important questions about the molecular nature and pleiotropy of such changes. We use *Drosophila sechellia*, a dietary specialist fruit fly that evolved to specialize on a single toxic host plant, *Morinda citrifolia*, as a model for adaptive phenotypic change and seek to determine the genetic basis of traits associated with host specialization in this species. The fruit of *M. citrifolia* is toxic to other drosophilids, primarily due to high levels of the defense chemical octanoic acid (OA), yet *D. sechellia* has evolved resistance to OA. Our prior work identified three *Osiris* family genes that reside in a fine‐mapped QTL for OA resistance: *Osiris 6* (*Osi6*), *Osi7*, and *Osi8*, which can alter OA resistance in adult *D. melanogaster *when knocked down with RNA interference suggesting they may contribute to OA resistance in *D. sechellia*. Genetic mapping identified overlapping genomic regions involved in larval and adult OA resistance in *D. sechellia*, yet it remains unknown whether *Osiris* genes contribute to resistance in both life stages. Furthermore, because multiple genomic regions contribute to OA resistance, we aim to identify other gene(s) involved in this adaptation. Here, we identify candidate larval OA resistance genes using RNA sequencing to measure genome‐wide differential gene expression in *D. sechellia* larvae after exposure to OA and functionally test identified genes for a role in OA resistance. We then test the *Osiris* genes previously shown to alter adult OA resistance for effects on OA resistance in larvae. We found that *Osi8* knockdown decreased OA resistance in *D. melanogaster* larvae. These data suggest that evolved changes in *Osi8 *could impact OA resistance in multiple life stages while *Osi6* and *Osi7* may only impact adult resistance to OA.

## 1 INTRODUCTION

*Drosophila sechellia* is a fruit fly endemic to the Seychelles islands that has evolved to specialize on a single host plant, *Morinda citrifolia* (Louis & David, 1986; Matute & Ayroles, 2014). This specialization is interesting because the ripe fruit of this plant is remarkably toxic to other *Drosophila* species (Jean‐Pierre, Legal, Moreteau, & Quéré, 1996; Legal, Chappe, & Jallon, 1994; Louis & David, 1986; R'Kha, Capy, & David, 1991). An abundance of carboxylic acids represent the majority of toxins identified in the fruit with octanoic acid (OA) making up 58% of all identified volatiles and is the primary component responsible for lethality in the fruit (Amlou, Pla, Moreteau, & David, 1997; Jean‐Pierre et al., 1996; Legal et al., 1994). *Drosophila sechellia* has evolved resistance to the toxins in *M. citrifolia* with many studies focused on evolved resistance to the primary toxin in the fruit OA.

Species in the *D. melanogaster* supercomplex are excellent models for understanding the genetics of adaptation and the evolutionary basis of toxin resistance as this clade contains both susceptible generalist (*D. melanogaster*, *D. mauritiana*, and *D. simulans*) and a derived resistant host specialist (*D. sechellia*) species. Recent evidence suggests *D. sechellia* has only recently undergone speciation from its sister species and is still capable of mating and producing viable and fertile offspring in crosses between *D. sechellia* and *D. simulans *allowing for genetic mapping of trait differences between species (R'Kha et al., 1991; Schrider, Ayroles, Matute, & Kern, 2018). While *D. sechellia* has evolved OA resistance across all life stages, OA is toxic to the generalist species in this clade across all stages of development (Jones, 1998, 2001; Legal, David, & Jallon, 1992; R'Kha et al., 1991). Several studies have investigated the genetic basis of OA resistance in *D. sechellia* adults (Amlou, Moreteau, & David, 1998a; Andrade López et al., 2017; Hungate et al., 2013; Jones, 1998; Peyser, Lanno, Shimshak, & Coolon, 2017; R'Kha et al., 1991) as well as in larvae (Amlou, Moreteau, & David, 1998b; Huang & Erezyilmaz, 2015; Jones, 2001); however, the specific genes involved in OA resistance and whether the same gene(s) are involved in OA resistance in multiple life stages remain unclear.

Preliminary genetic analyses of evolved differences in resistance to *Morinda* fruit involved determining regions of the genome responsible for variation in this trait between resistant species *D. sechellia *and susceptible sister species *D. simulans*. R'Kha *et al*. estimated three to five resistance factors contribute to this variation using a biometric approach analyzing adult and embryonic survival (R'Kha et al., 1991). Building on this work, genetic markers were used for initial mapping of OA resistance factors in *D. sechellia* adults (Jones, 1998) and later in *D. sechellia* larvae (Jones, 2001). Similar to the findings of R'Kha et al. (1991), Jones found that at least five loci were involved in adult *D. sechellia* resistance and approximated their relative size of effect and location in the genome. The region of largest effect was identified on chromosome 3R that explained approximately 15% of the variation in adult OA resistance between susceptible species *D. simulans* and resistant species *D. sechellia*. Initial mapping of larval OA resistance (measuring survival from egg to adult) found three loci that contribute to resistance during these developmental stages. Again, a region on chromosome 3R was found to harbor a resistance factor of the greatest effect (Jones, 1998, 2001, 2005). This work suggested that there was a partial but not complete overlap in the QTL that contributes to OA resistance in different developmental stages. Because the QTL maps generated in these studies were low resolution, it remained a challenge to determine the degree to which genetic basis for OA resistance in *D. sechellia *was the same or different across life stages.

Substantially increased mapping resolution for both adult (Hungate et al., 2013) and larval (Huang & Erezyilmaz, 2015) OA resistance has helped to narrow the focus for functional interrogation of the genes underlying OA resistance, but also yielded additional challenges. Huang & Erezyilmaz performed QTL analysis in postembryonic stages of *D. sechellia* using more than 400,000 markers. This work greatly increased the resolution of OA resistance regions associated with molting during larval development (Huang & Erezyilmaz, 2015). Interestingly, the QTL map generated in this study differed from that reported by Jones (2001), possibly because of the limited resolution of the earlier study or because of differences in the method for determining larval OA resistance (survival from L2 larvae to pupae formation was measured in Huang & Erezyilmaz, 2015 vs. egg‐to‐adult survival in Jones, 2001).

High‐resolution introgression mapping of adult OA resistance identified a small region that underlies the major effect QTL identified previously on chromosome 3R (Jones, 1998) that narrowed this resistance region to a 170‐kb window containing only 18 genes (Hungate et al., 2013). The genes in this region have a variety of molecular functions including several odorant binding proteins (*Obps*) and nine genes in the *Osiris* family. These 18 genes were functionally tested by Andrade López et al. (2017) using RNA interference (RNAi) in *D. melanogaster* adults to identify candidate resistance genes. Three genes in the *Osiris* family, *Osiris 6 *(*Osi6*), *Osi7*, and *Osi8*, were found to significantly decrease adult resistance to OA when their expression was ubiquitously knocked down (Andrade López et al., 2017). This discovery, along with recent findings from other researchers, points to the intriguing, yet enigmatic, *Osiris* gene family influencing evolved toxin resistance. For example, Yassin et al. (2016) conducted a population genomics scan between an island specialist population of *D. yakuba* that recently specialized on *Morinda citrifolia* and a mainland generalist population of the same species and found that one of the strongest differentiation peaks mapped to the *Osiris* cluster on chromosome 3R (Yassin et al., 2016) possibly illustrating a case of parallel evolution through this locus. Only one other *Osiris* gene, *Osi21*, has been functionally tested in *D. melanogaster* and loss‐of‐function alleles of this gene shift membrane balance between late endosomes and lysosomes facilitating degradation of endocytosed rhodopsin in eye cells (Lee, Song, & Hong, 2013). This finding is interesting as it suggests *Osiris* genes may be involved in detoxification. Although little is known about the molecular function of all other *Osiris* genes, sequence analysis and similarity between *Osiris* genes suggests they may serve similar functions in membrane homeostasis of the endomembrane system (Dorer, Rudnick, Moriyama, & Christensen, 2003; Shah, Dorer, Moriyama, & Christensen, 2012). In addition to a potential role in detoxification, *Osiris* genes have also been associated with chitin and cuticle development, suggesting an additional possible mechanism of toxin resistance mediated through bolstered physical barriers (Lanno et al., 2017; Smith, Morandin, Noureddine, & Pant, 2018). However, the molecular mechanism of *Osiris* effects on OA resistance remains unknown.

While our recent work identified the first genes that can alter OA resistance in adults, less progress has been made in identifying the specific genes underlying OA resistance in *D. sechellia *larvae. Methods of examining *D. sechellia *larval OA resistance vary among previous studies. Amlou et al. (1998b) studied larval resistance by calculating the proportion of adults emerging from pupae after larvae were exposed to varying concentrations of OA (Amlou et al., 1998b). Similarly, Jones (2001) determined egg‐to‐adult resistance by dividing the number of emerging adults by the number of eggs laid in each OA environment, calculating percentage survival (Jones, 2001). Huang and Erezyilmaz (2015) assessed larval resistance by calculating the percentage of larvae that formed puparia after being transferred into environments of varying levels of OA during the second larval instar and found that their assay was primarily measuring mortality associated with OA exposure during molting (Huang & Erezyilmaz, 2015). While these studies demonstrate OA resistance through postembryonic development, our study specifically focused on larval resistance to acute OA toxicity during a single larval stage to avoid the complications associated with the possibility that each stage could involve evolutionary changes in different genes, a possibility supported by the nonoverlap in the QTL identified for different studies of larval OA resistance (Huang & Erezyilmaz, 2015; Jones, 2001).

Here, we present an investigation of the genetic basis of OA resistance in *D. sechellia* larvae by addressing two main hypotheses. First, candidate larval OA resistance genes can be discovered through identification of differential gene expression responses to larval OA exposure using RNA sequencing (RNA‐seq). This approach was successful in a previous study investigating OA resistance in adults followed by associated functional genetic testing (Andrade López et al., 2017; Lanno et al., 2017). Second, that larval OA resistance may involve the same genes or a subset of those genes involved in adult OA resistance as suggested by Jones (2001). While it is clear there is nonoverlap in the adult and larval QTL maps, we are specifically interested in whether *Osiris* gene(s) contribute to both developmental stages given their identification in adult OA resistance. To do this, we performed genome‐wide differential gene expression testing between control and OA‐exposed *D. sechellia* larvae using RNA‐seq and functionally tested candidate OA resistance genes using RNA interference (RNAi) in *D. melanogaster* with acute larval OA resistance assays. In addition, we performed functional testing of *Osiris* genes (*Osi6*, *Osi7*, and *Osi8*) previously shown to alter OA sensitivity in adult flies to determine whether the same genes may be involved in both larval and adult stages.

## 2 METHODS

### 2.1 Fly strains and maintenance

Strains of four *Drosophila* species were used in this study: *D. melanogaster *(14021‐0231.36, *w^1118^*, and a GeneSwitch‐GAL4 driver (*Tubulin*‐P[Switch]) (Wang, O'Malley, & Tsai, 1994))*, D. sechellia *(14021‐0428.25), *D. simulans* (14021‐0251.195), and *D. mauritiana *(14021‐0241.60). Additional *D. melanogaster* UAS‐RNAi lines from the Vienna *Drosophila* UAS‐RNAi Center (Dietzl et al., 2007) (VDRC# 102392, 44545, 8475, 5753, 109528, 15590, & 110406; for full genotypes, see Supporting Information Table S1) were used. All flies were reared on cornmeal medium using a 16:8 light:dark cycle at 20°C. Wandering‐stage (WS) larvae were collected and analyzed from these strains and crosses made from them for RNAi experiments.

### 2.2 Octanoic acid resistance assays

Wandering‐stage *D. melanogaster*, *D. sechellia*, *D. simulans*, and *D. mauritiana* larvae were collected from control bottles containing cornmeal medium and placed into a 35‐mm petri dish containing 0.6 g *Drosophila* medium (Carolina Biological Supply Company Formula 4‐24^®^), 2 ml H_2_O, and 31.2 µl OA (1.2% OA by weight). A concentration of 1.2% OA was chosen as it has been previously observed to knock down ~50% of *D. melanogaster* adult flies over a 1‐hr exposure period (Andrade López et al., 2017) allowing us to make comparisons between larval and adult survival at this OA concentration. The number of individual larvae “knocked down” was determined every 5 min for 60 min. Larvae were determined to be “knocked down” when they showed no apparent signs of life (turned brown or black, maintained rigidity, showed no peristalsis, and displayed no movement when nudged with blunt forceps). Larval survivorship was averaged across eight biological replicates at a density of 10 larvae per dish by determining the number of larvae knocked down in each experiment (*n* = 80 for each species). Adult OA resistance assays were performed as described in Andrade López et al. (2017).

### 2.3 Cox proportional hazards regression model and analysis

The *coxph* command in the survival package (Therneau, 2015; Therneau & Grambsch, 2000) from the R library (R Core Development Team, 2010) was used to measure differences in survival among *D. melanogaster *supercomplex species exposed to control or OA environments over the period of 1 hr. The *coxph* function was used as follows, where X can be one of two explanatory variable options: (1) species * stage or (2) ± RU486, and Y is the data used (differs for model option 1 vs. model option 2):

coxph(Surv(Time, Status)∼X, data=Y, ties=c(``efron''))

### 2.4 RNA sequencing and library prep

*Drosophila sechellia *(14021‐0428.25) flies were reared on cornmeal medium using a 16:8 light:dark cycle at 20°C. Wandering F_1_ larvae (stage L3) were collected and exposed to either control food or food containing 0.2% OA for an exposure period of 1 hr. Following exposure, larvae were snap‐frozen in liquid nitrogen and stored at −80°C until RNA extraction. RNA was extracted from a homogenate of 10 whole larvae using the Promega SV total RNA extraction system with modified protocol (Promega; Coolon, Webb, & Wittkopp, 2013). Three biological replicates were produced for each treatment for a total of six sequencing libraries. Five microliters of RNA from each extraction was checked via gel electrophoresis to confirm successful RNA extraction. RNA quality control (BioAnalyzer and NanoDrop), library preparation (TruSeq mRNA library preparation kit), and RNA sequencing (Illumina HiSeq‐4000, H4K Single End 50 Cycle) were performed by the University of Michigan Medical School DNA Sequencing Core.

### 2.5 Differential gene expression testing using RNA sequencing

An RNA‐seq pipeline was performed in Galaxy (Afgan et al., 2016). All read files were checked for quality using FASTQC (Andrews, 2010). Reads were mapped to the *D. sechellia* genome using Bowtie 2 (Langmead & Salzberg, 2012) and the current genome file available from Ensembl at the time of this analysis: Drosophila_sechellia.GCA_000005215.1.dna.toplevel.fa (Yates et al., 2016). Gene expression quantification and differential expression analysis was performed with Cuffdiff (Trapnell et al., 2013, 2010) using the aforementioned genome file and the most recent gene file available from Ensembl: Drosophila_sechellia.GCA_000005215.1.34.gff3 (Yates et al., 2016). Cuffdiff was run with geometric library normalization and bias correction was performed using the reference genome sequence (Drosophila_sechellia.GCA_000005215.1.dna.toplevel.fa). Visualization of our gene expression data was performed in R (R Development Core Team 2011). Gene name orthologs were downloaded from FlyBase (Attrill et al., 2016) for all *D. sechellia* genes to transform differentially expressed gene names into *D. melanogaster* namespace for gene ontology (GO) term enrichment. GO term enrichment analysis was performed using the Gene Ontology Consortium (Ashburner et al., 2000; Blake, Christie, & Dolan, 2015). Differentially expressed genes were then cross‐referenced with genes residing in larval OA resistance QTLs derived from *D. sechellia* and *D. simulans* described by Huang and Erezyilmaz (2015, Supporting Information Files S4 to S12).

### 2.6 Stage‐specific RNAi knockdown of candidate OA resistance genes

Larvae used in the RNAi knockdown study were generated by crossing 20 virgin *Tubulin*‐P[Switch]‐GAL4 females to 20 UAS‐RNAi males per bottle, set up in experimental and control pairs for each gene knocked down. Functional gene testing was performed using the Gene‐Switch RNAi system involving a hormone‐induced *Tubulin*‐P[Switch] GAL4 driver consisting of a modified chimeric GAL4 gene (Gene‐Switch) that encodes the GAL4 DNA binding domain, the human progesterone receptor ligand‐binding domain, and the activation domain from human protein *p65*. The chimeric molecule only becomes active in the presence of the synthetic antiprogestin mifepristone (RU486). When active, the molecule binds to the upstream activating sequence (UAS) to activate transcription of the RNA hairpin, resulting in knockdown of gene expression only in the presence of RU486 (Andrade López et al., 2017; Osterwalder, Yoon, White, & Keshishian, 2001; Roman, Endo, Zong, & Davis, 2001). Once stage L1 larvae were visible in bottles, adult flies were transferred to new bottles for further egg‐laying. At this point, either 1 ml of a stock solution of 10 mg/ml mifepristone (RU486 Sigma, St. Louis) in 100% EtOH diluted to a final concentration of 10 µg/ml (knockdown) or 1 ml of 10 µl/ml EtOH (control) was added directly to the bottles to compare genetically identical F_1_ offspring in OA resistance assays. Larvae experienced each treatment for at least 24 hr before OA resistance assays, and throughout the assays. The 1.2% OA resistance assay described earlier was then performed on RNAi knockdown larvae, with a final concentration of 10 µg/ml RU486 or EtOH also being mixed into food for each treatment.

### 2.7 Quantifying gene expression with qRT‐PCR

Larval gene expression levels were measured in *D. sechellia* and *D. simulans* for *Osi6*, *Osi7*, *Osi8*, and the housekeeping gene *alpha‐tubulin *(*αTub84B*) using quantitative reverse transcriptase PCR (qRT‐PCR) as done previously (Andrade López et al., 2017). Larvae were collected from bottles containing cornmeal medium and placed into a 35‐mm petri dish containing either control food (0.6 g *Drosophila* medium, 2 ml H_2_O per dish) or food containing 0.2% OA (0.6 g *Drosophila* medium, 2 ml H_2_O, 5.2 µl OA per dish). A concentration of 0.2% OA was chosen for future gene expression studies in order to obtain expression levels for susceptible sister species *D. simulans* because further increases in OA concentration cause acute mortality. Larvae were exposed to each environment for a period of 1 hr. After the exposure period, larvae were immediately collected and snap‐frozen in liquid nitrogen, placed on dry ice, and stored at −80°C until use. RNA was extracted using the Promega SV Total RNA Isolation System with modified protocol (Promega, Coolon et al., 2013). cDNA synthesis was performed using total RNA with T(18) primers and SuperScript II (Invitrogen) according to manufacturer recommendations. qRT‐PCR was performed in an Applied Biosystems StepOne Plus thermocycler using Applied Biosystems PowerUp SYBR Green reagents. For each sample, Applied Biosystems PowerUp SYBR Green Master Mix (20 µl) was mixed with 0.4 µl GoTaq DNA Polymerase, 7.6 µl nuclease‐free water, and 8 µl cDNA and split into four reactions containing 9 µl each. Once split, gene‐specific primers (Supporting Information Table S2) were added (0.5 µl each forward and reverse) for a total volume of 10 µl per reaction. Cycling conditions for PCR were the same for all genes except for different annealing temperatures: 50°C for 2 min followed by 95°C for 2 min, followed by 50 cycles of 95°C for 15 s, annealing temp (56°C for *Osi6*, *Osi7*, and *Osi8*, 63°C for *αTub84B*) for 30 s, and 72°C for 30 s. Melt curves were generated for each reaction to ensure specificity. Threshold cycle (C_T_) values were generated for each reaction based on entry into log phase amplification during PCR. For *Osi6*, *Osi7* and *Osi8*, ΔC_T_ values were generated by correcting each against the housekeeping gene *αTub84B*. Three biological replicates were run for each sample type, and *t* tests were performed to evaluate statistical significance. For comparisons between flies reared on control food and food containing OA, ΔΔC_T_ values were generated by subtracting control – OA for each sample and *t* tests were performed testing against 0 (Andrade López et al., 2017).

### 2.8 DNA coding and protein sequence analyses of *Osi8*

DNA coding sequences (CDS) for *Osi8* were downloaded from FlyBase (Attrill et al., 2016) for *D. melanogaster*, *D. sechellia*, and *D. simulans*. Clustal Omega (Goujon et al., 2010; McWilliam et al., 2013; Sievers et al., 2011) was used to align DNA CDS and translated protein sequences in order to determine synonymous and nonsynonymous differences between these species. To map the location of nonsynonymous changes within the *Osi8* protein in *D. sechellia*, comparisons were made between its protein coding sequence and sequence logos of the conserved domains of *Osiris* proteins determined by multiple sequence alignments in *D. melanogaster *(Shah et al., 2012). Signal peptide and transmembrane predictions for the *D. sechellia Osi8* protein were made with Phobius (version 1.01) (Käll, Krogh, & Sonnhammer, 2007) and SOSUIsignal (Gomi, Sonoyama, & Mitaku, 2004). Discrimination of signal peptides from transmembrane regions was performed with SignalP (server version 4.1) (Petersen, Brunak, Heijne, & Nielsen, 2011). To further analyze *Osi8* protein CDS variation, we performed additional *Osi8* alignments using Clustal Omega on other *Drosophila* species including *D. mauritiana*, *D. suzukii*, *D. erecta*, *D. yakuba*, *D. ananassae*, *D. pseudoobscura*, *D. persimilis*, *D. willistoni*, *D. virilis*, *D. mojavensis*, and *D. grimshawi*, as well as non‐Drosophila dipterans including *Aedes aegypti*, *Lucilia cuprina*, *Anopheles darlingi*, *Anopheles gambiae*, *Culex quinquefasciatus*, and *Glossina morsitans*, and nondipteran insects including *Danaus plexippus*, *Nasonia vitripennis*, and *Tribolium castaneum* downloaded from FlyBase (Attrill et al., 2016).

### 2.9 Investigating *Osi8* CDS variation in multiple *D. sechellia* genotypes using DNA variant calling

Paired‐end DNA sequencing files from 23 different wild‐caught *D. sechellia* genomes from the Seychelles islands of Mahe (*n* = 7), Praslin (*n* = 7), La Digue (*n* = 7), and Marianne (*n* = 2) were downloaded from NCBI's Short Read Archive (BioProject number PRJNA395473) (Schrider et al., 2018). Each read file was mapped to a fasta file containing the *D. sechellia Osiris 8* DNA CDS using Bowtie 2 (Langmead & Salzberg, 2012). Aligned reads were then assessed for within‐species variation of the *Osi8* allele using the Naïve Variant Caller (Blankenberg et al., 2014) in Galaxy using the *D. sechellia Osi8* DNA CDS downloaded from FlyBase as the reference line (Attrill et al., 2016).

## 3 RESULTS

### 3.1 Quantifying OA resistance among *Drosophila melanogaster* supercomplex species larvae

**Figure 1:**
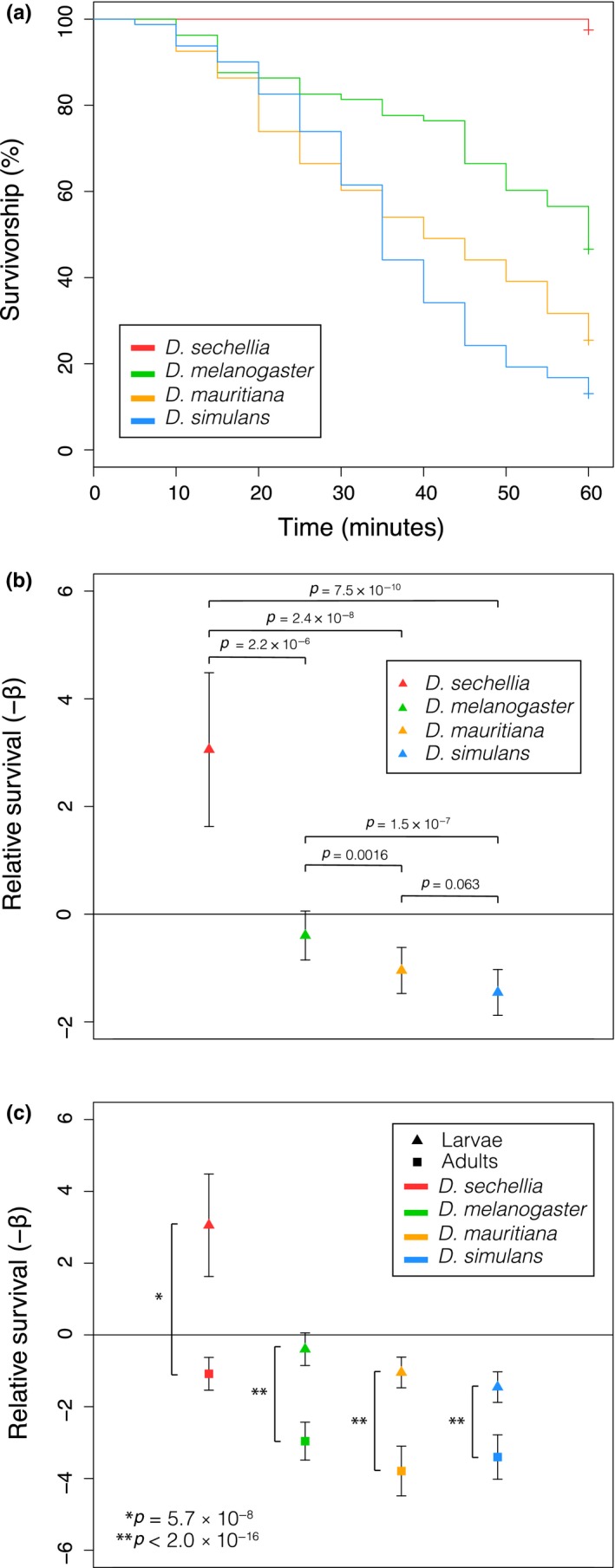
Measuring OA resistance and relative survival between species and life stages. (a) Survival curves from a 1.2% OA resistance assay including *D. sechellia* (red), *D. melanogaster* (green), *D. mauritiana* (orange), and *D. simulans* (blue) larvae (*n* = 80 for all species). (b) Relative survival of larvae from each species relative to a baseline from the *D. melanogaster w^1118^* line shown as −β from a Cox regression model. Error bars represent 1.96 standard error (*SE*) relative to the baseline, and *p* values represent significant differences in survival from Cox regression analyses of the survival curves in (a). (c) Relative survival of adults and larvae from each species relative to the same *D. melanogaster w^1118^* baseline. Error bars represent 1.96 *SE* relative to the baseline, and *p* values represent significant differences in survival between adults and larvae of the same species

To test whether species in the *Drosophila melanogaster *supercomplex differ in resistance to OA, we quantified larval survivorship over 1 hr of exposure to 1.2% OA in food. After 1‐hr exposure, *D. sechellia* larvae showed the greatest survivorship (98%), followed by *D. melanogaster* (46%), *D. mauritiana* (25%), and *D. simulans* (13%) (*n* = 80 for all species). Using the Cox proportional hazards statistical model (Cox, 1972; Fox, 2008) to analyze the survivorship curves, *D. sechellia* showed significantly greater survival than all three other species over the exposure period (*p* < 2.2 × 10^−6^ in all cases; Figure 1a). *Drosophila melanogaster *showed significantly greater survival than both *D. mauritiana* (*p* = 0.0016) and *D. simulans* (*p* = 1.5 × 10^−7^). No significant difference in survival was found between *D. simulans* and *D. mauritiana* (*p* = 0.063). While a single strain was used to represent each species, prior work found that between‐species differences far exceeded any observed within‐species variation among strains, and therefore, the observed differences primarily represent fixed species differences (Andrade López et al., 2017). Regression coefficients from Cox proportional hazards analyses were used to determine the relative survival (−β) of each species when exposed to OA. All species were compared to a reference *D. melanogaster* line (*w^1118 ^v60000*). This analysis reflects the patterns observed in the survival curves with *D. sechellia* having the greatest relative survival (−β = 3.056), *D. melanogaster* (−β = −0.397) having intermediate survival, and *D. mauritiana* (−β = −1.046), and *D. simulans *having the poorest relative survival (−β = −1.454) (Figure 1b). All lines analyzed showed significantly different survival compared to the reference *D. melanogaster w^1118^* line (*p* < 2.7 × 10^−5^ in all cases) except the other *D. melanogaster* (14,021–0,231.36) line tested (*p* = 0.087).

### 3.2 Larval survival over a sixty‐minute exposure period to 1.2% OA was much greater than adult survival

While both larval and adult resistance to OA have been quantified, a direct comparison of the resistance of these two life stages had not yet been made. To do this, we compared relative survival of larvae and adults in response to OA exposure. WS larvae of all four species used in this analysis showed significantly greater survival than their adult counterparts over 1‐hr exposure to 1.2% OA mixed into food (Figure 1c). Regression coefficients for relative survival were determined using a multivariate Cox proportional hazards model. Overall, we found that there was a significant main effect of stage (−β = 1.75, *p* < 2 × 10^−16^), species (−β = −0.69, *p* < 2 × 10^−16^) and a significant interaction between stage and species (−β = 0.24, *p* = 0.011). In specific contrasts between larval and adult survival, we found *D. sechellia* (−β = −1.083), *D. melanogaster* (−β = −2.96), *D. mauritiana* (−β = −3.792), and *D. simulans* (−β = −3.402) showed a similar rank order in larvae as in adults, but all adults had significantly poorer survival than larvae (*p* < 5.7 × 10^−8^ in all cases). The significant interaction between species and stage is driven by the difference in the magnitude of the difference between larval and adult survival among the tested species (Figure 1c).

### 3.3 Genome‐wide differential expression testing using RNA sequencing to identify candidate genes involved in OA resistance

**Figure 2:**
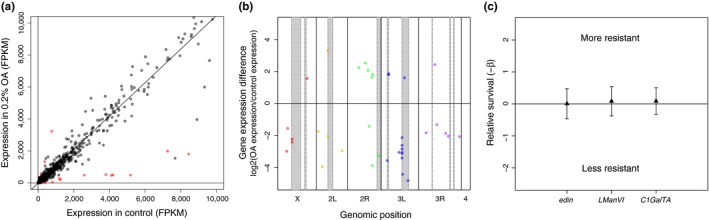
RNA‐seq analysis of larval gene expression response to OA and functional tests of candidate genes. (a) Scatter plot of all differentially expressed genes in *D. sechellia* larvae upon exposure to 0.2% OA in fragments per kilobase of transcript per million mapped reads (FPKM) are shown (red = significant; black = n.s.). (b) Significantly differentially expressed genes are plotted according to chromosomal position in *D. melanogaster* (red = genes on the X chromosome; orange = 2L; green = 2R; blue = 3L; purple = 3R). No significantly differentially expressed genes were found on the fourth chromosome. Gray‐shaded areas represent QTL peak ranges for larval OA resistance described by Huang and Erezyilmaz (2015). (c) Relative survival of *D. melanogaster *RNAi larvae targeting candidate OA resistance genes revealed by RNA‐seq. There was no observed mortality associated RNAi in the absence of OA exposure (data not shown). Error bars represent *SE*

We next sought to identify candidate genes that may contribute to evolved larval OA resistance through induced changes in gene expression. Recent QTL mapping data have narrowed the regions containing larval OA resistance factors (Huang & Erezyilmaz, 2015); however, QTL analyses typically yield confidence intervals that are too large to reveal specific candidate genes. Prior work has shown that genes with environmentally induced expression frequently contribute to fitness in that environmental context (Coolon, Jones, Todd, Carr, & Herman, 2009). Therefore, to further reduce the number of candidate genes, we used RNA‐seq for differential gene expression analysis between *D. sechellia* larvae fed a control diet and larvae exposed to 0.2% OA mixed into food. This analysis revealed 44 significantly differentially expressed genes (Figure 2a) including one overrepresented 5S rRNA (RF00001), which was removed from further analysis. Of the remaining 43 genes, 39 have annotated orthologs in *D. melanogaster* allowing GO term enrichment as well as providing a list of candidate larval OA resistance genes for future functional testing (Supporting Information Tables S3 and S4). GO term analysis revealed nine significantly enriched biological process terms, all relating to immune and bacterial defense responses (Supporting Information Table S5). Using chromosomal coordinates for these genes in *D. melanogaster*, we plotted the location of each gene according to its position on each chromosome and its relative expression level (Figure 2b). We then compared the significantly differentially expressed genes determined by RNA‐seq to genes within QTL peak ranges and candidate gene lists provided by mapping data from prior studies (Huang & Erezyilmaz, 2015, supplementary material) and identified 17 genes residing within these QTLs, including three genes with annotated orthologs in *D. melanogaster* which were then functionally tested for larval OA resistance using RNAi in *D. melanogaster* larvae.

### 3.4 Functional testing of candidate larval OA resistance genes revealed by RNA‐seq

RNA sequencing revealed candidate genes for functional testing in OA resistance assays. RNAi knockdown was first performed using candidate genes revealed by RNA‐seq on genetically identical WS larvae using the hormone‐inducible Gene‐Switch system (Andrade López et al., 2017) in *D. melanogaster*. We observed no mortality associated with knockdown in the absence of OA exposure. By crossing each RNAi‐UAS line to the *Tubulin*‐P[Switch] GAL4 line and comparing sibling offspring from this cross with and without gene knockdown (+RU486 knockdown or +EtOH control), we found no significant differences in OA resistance after knockdown of *edin* (*p* = 0.99), *LManVI* (*p* = 0.85), or *C1GalTA* (*p* = 0.83; Figure 2c). We next functionally tested *Osiris* genes previously described to alter adult OA resistance by performing RNAi during this larval stage.

### 3.5 Stage‐specific RNAi knockdown of *Osi8* decreases larval resistance to OA

Prior lower resolution mapping data suggested regions of chromosome 3R contribute to larval resistance to OA. Recent mapping with greater resolution suggests the cluster of *Osiris* genes on 3R are not contained within QTL peaks mapped for larval resistance associated with OA exposure from second larval instar to pupation (Huang & Erezyilmaz, 2015; Hungate et al., 2013; Jones, 1998, 2001). Because altered *Osiris* gene expression or function likely confers resistance to adult *D. sechellia *flies, QTL mapping data in prior work disagree about the contribution of some loci (Huang & Erezyilmaz, 2015; Jones, 2001), and the prior work likely measured OA resistance associated with molting periods, we wanted to test whether *Osi6*, *Osi7*, and *Osi8* are involved in larval resistance to acute exposure in wandering mid‐stage L3 larvae. All three genes have been previously shown to alter OA resistance when knocked down in *D. melanogaster* adults (Andrade López et al., 2017), so we conducted stage‐specific RNAi knockdowns of *Osi6*, *Osi7*, and *Osi8* and included *Osi5* as a control as it has not been previously implicated in OA resistance.

**Figure 3:**
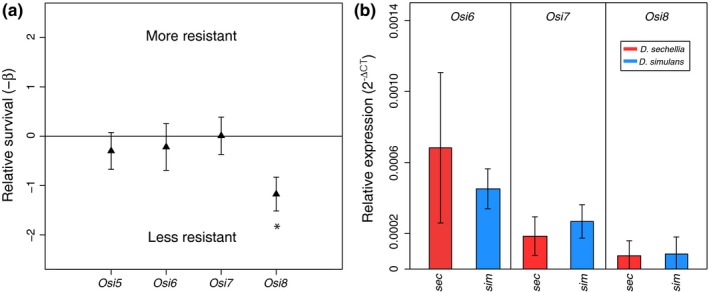
Stage‐specific RNAi knockdowns of *Osiris* genes. Relative survival of RNAi larvae targeting *Osiris* genes associated with OA resistance and corresponding expression analyses. (a) Relative survival of *D. melanogaster* RNAi larvae targeting *Osiris* genes. There was no observed mortality associated RNAi in the absence of OA exposure (data not shown). Error bars represent *SE*, asterisk represents a significant difference in survival between control and RNAi lines (*p* = 0.00061). (b) Relative gene expression was measured in WS larvae with qRT‐PCR targeting *Osi6*, *Osi7*, and *Osi8*. Expression levels of *Osi6*, *Osi7*, and *Osi8* genes measured in *D. sechellia* (red), and *D. simulans* (blue). Normalized relative expression is shown (2^−ΔCT^), and error bars represent *SE*

RNAi knockdown of *Osiris* genes was performed on WS *D. melanogaster* larvae using the Gene‐Switch hormone‐inducible system, and we observed no mortality associated with knockdown in the absence of OA exposure. By crossing each RNAi‐UAS line to the *Tubulin*‐P[Switch] GAL4 line and comparing sibling offspring from this cross with and without gene knockdown (+RU486 knockdown or +EtOH control), we found that knockdown of *Osi8* significantly reduced OA resistance when compared to sibling offspring not receiving the RU486 hormone (*p* = 0.00061) as well as the *D. melanogaster w^1118^* reference line (*p* = 0.017) (Figure 3a), similar to that observed in adults. Unlike that observed in adults, knockdown of *Osi6* (*p* = 0.65) and *Osi7* (*p* = 0.98) did not significantly alter OA resistance (Andrade López et al., 2017). We also tested *Osi5* knockdown larvae as a negative control and found no changes in OA sensitivity (*p* = 0.42). Because gene expression changes of *Osi6* and *Osi7* were previously observed and thought to contribute to adult OA resistance, we next investigated larval expression of *Osi6*, *Osi7*, and *Osi8*.

### 3.6 *Osiris 6*, *Osi7*, and *Osi8* gene expression is similar in *D. simulans* and *D. sechellia* larvae

To investigate levels of gene expression and possible differences between adult and larval expression of *Osiris* genes, we used qRT‐PCR to analyze normalized mRNA levels in *D. sechellia* and *D. simulans* larvae (Figure 3b). Because we chose WS larvae to use in our analysis, a stage of rapid developmental change, some variability in gene expression was expected. Notably, *D. sechellia* larvae had *Osi6*, *Osi7*, and *Osi8* expression levels that were not significantly different from susceptible species *D. simulans*, in contrast to the much higher standing levels of expression observed in* D. sechellia* adults than *D. simulans* adults for these genes (Andrade López et al., 2017). There were no significant differences in expression of *Osi8* among any of the species analyzed similar to the gene expression pattern observed in adults (Andrade López et al., 2017), strengthening the hypothesis that protein sequence changes in the *D. sechellia* allele of *Osi8* may be contributing to OA resistance rather than differences in the expression of this gene (Andrade López et al., 2017). In our larval RNA‐seq analysis, *Osi6*, *Osi7*, and *Osi8* did not show significant differential expression in *D. sechellia* larvae upon 0.2% OA exposure, similar to results observed in adults, and these results were confirmed with qRT‐PCR (Supporting Information Figure S1).

### 3.7 DNA and protein coding sequence analyses of *Osi8*

**Figure 4:**
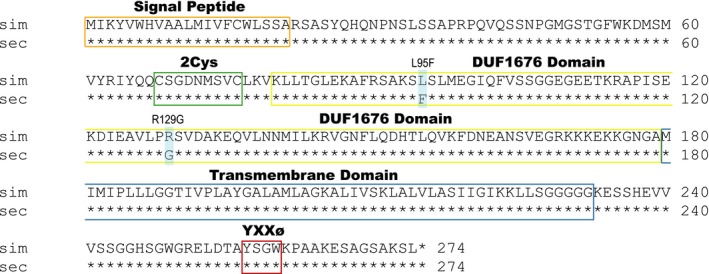
Mutations in the *Osi8* protein coding sequence of *D. sechellia*. Aligned protein coding sequences (CDS) of *D. simulans* and *D. sechellia* are shown. Asterisks represent conserved amino acids. Boxes outline conserved regions of *Osiris* genes including the signal peptide (orange), 2Cys region (green), duf1676 domain (yellow), and putative transmembrane domain (blue), along with a YXXø signaling motif (red). Transparent blue rectangles highlight protein CDS mutations (L95F & R129G)

To identify any mutations that may confer resistance through changes in protein function, the coding sequences of *Osi8* in *D. melanogaster*, *D. sechellia*, and *D. simulans* were downloaded from FlyBase (Attrill et al., 2016) and aligned with Clustal Omega (Goujon et al., 2010; McWilliam et al., 2013; Sievers et al., 2011). This analysis revealed two nonsynonymous changes in the *D. sechellia* protein (L95F and R129G; Figure 4) (Andrade López et al., 2017). *Osiris* proteins exhibit four conserved domains across members of the gene family. These domains include a two‐cysteine region, a domain of unknown function, duf1676 (Pfam family: PF07898; Finn et al., 2016), a putative transmembrane domain, and a region containing an AQXLAY motif (Shah et al., 2012). Both amino acid altering mutations occur in the domain of unknown function, duf1676. Multiple bioinformatics analyses (see Section 2) confirm predicted domains of *Osi8* including an endoplasmic reticulum signal peptide sequence and two transmembrane helices (Supporting Information Figure S2, Supporting Information Table S6).

To investigate whether the *Osi8* mutations observed in *D. sechellia* (L95F & R129G) are evolved changes unique to this species additional *Osi8* alignments involving other Drosophila species including *D. mauritiana*, *D. suzukii*, *D. erecta*, *D. yakuba*, *D. ananassae*, *D. pseudoobscura*, *D. persimilis*, *D. willistoni*, *D. virilis*, *D. mojavensis*, and *D. grimshawi*, as well as non‐*Drosophila* dipterans including *Aedes aegypti*, *Lucilia cuprina*, *Anopheles darlingi*, *Anopheles gambiae*, *Culex quinquefasciatus*, and *Glossina morsitans*, and nondipteran insects including *Danaus plexippus*, *Nasonia vitripennis*, and *Tribolium castaneum* were performed. These alignments revealed all 13 other *Drosophila* species, six non‐Drosophila dipterans, and three nondipteran insects all contain a conserved arginine residue at the 129 position and the derived glycine residue in this position is unique to *D. sechellia *(Supporting Information Figure S3a). The other mutation, L95F, was only observed in one other distantly related *Drosophila* species, *D. grimshawi*, while all other species analyzed showed a conserved leucine at this position (Supporting Information Figure s3b).

To additionally investigate within‐species variation of the mutations in *Osi8* in *D. sechellia*, DNA variant calling was performed on paired‐end DNA sequencing files from 23 different wild‐caught *D. sechellia* genomes from the Seychelles islands of Mahe (*n* = 7), Praslin (*n* = 7), La Digue (*n* = 7), and Marianne (*n* = 2) (BioProject number PRJNA395473) (Schrider et al., 2018). This analysis revealed both the L95F and R129G mutations are conserved across all 23 strains of *D. sechellia* with no within‐species variation observed at these positions demonstrating the derived allele of *Osi8* containing the two nonsynonymous mutations is fixed in *D. sechellia* (Supporting Information Table S7).

## 4 DISCUSSION

### 4.1 RNA‐seq and Functional testing of candidate OA resistance genes

We demonstrate evolved OA resistance in *D. sechellia* larvae compared to the larvae of other *D. melanogaster* supercomplex species using an OA resistance assay. In a between‐species survival comparison, we found that larvae of the *D. melanogaster* supercomplex species showed a comparable rank order of OA resistance to that observed in adults, with *D. sechellia* larvae conveying the greatest resistance and almost 100% survival, followed by intermediate resistance in *D. melanogaster*, and poor survival in *D. simulans* and *D. mauritiana* when exposed to 1.2% OA (Andrade López et al., 2017; Jones, 1998). We showed that larvae were significantly more resistant to the toxic effects of high levels of OA over a 60‐min exposure period than adult flies. Within‐species comparisons of adult and larval resistance showed that larvae of the same species were more resistant than their adult counterparts across all species analyzed.

Differential gene expression analysis using RNA‐seq to find genes differentially expressed between control and OA environments in *D. sechellia* larvae revealed 43 significantly differentially expressed genes. Of these, three genes with annotated orthologs in *D. melanogaster *resided within QTL peaks for larval OA resistance (*edin*, *LManVI*, and *C1GalTA*). We functionally tested these candidate OA resistance genes and observed no significant changes in OA sensitivity upon knockdown of these genes with RNAi, suggesting the induced changes in expression are not related to OA resistance. It remains possible that the other differentially expressed genes identified in this study may represent novel genes contributing to OA resistance in *D. sechellia*. Future studies focusing on the role of these genes in OA resistance are warranted. Additionally, genes associated with toxin resistance may have evolved through changes in constitutive gene expression levels that would not be detected by differential gene expression analysis in response to OA exposure. GO enrichment analysis revealed two main significant biological process terms: defense response to gram‐positive bacterium (*p* = 2.15 × 10^−4^), and humoral immune response (*p* = 3.92 × 10^−3^), terms that were also enriched for *D. sechellia *adults exposed to OA (Lanno et al., 2017), suggesting a possible role for immunity‐associated genes in OA resistance, though this is yet to be investigated. We then showed that knockdown of *Osi8* in *D. melanogaster* larvae significantly decreased OA resistance, consistent with previous findings in *D. melanogaster* adults. Alternatively, we also showed that gene expression levels of *Osi6* and *Osi7* in *D. sechellia* larvae are not significantly different from those of susceptible sister species *D. simulans*, a finding different from adult *D. sechellia* where gene expression levels are extremely high relative to susceptible sister species (over 70 times higher expression of *Osi6* and *Osi7* in adult *D. sechellia* vs. *D. simulans*, Andrade López et al., 2017). These gene expression results are consistent with our larval RNAi results as *Osi6* and *Osi7* did not alter OA resistance when knocked down in *D. melanogaster *larvae. Together these results may suggest that both protein coding mutations (*Osi8*, observed in adults and larvae) as well as regulatory mutations affecting expression (*Osi6* and *Osi7*, observed only in adults) of *Osiris* genes may contribute to variation in OA resistance. Our results also suggest *Osiris* genes could play a larger role in adult resistance than larval resistance. These findings are consistent with the hypothesis that *D. sechellia *larval resistance to OA may use only a subset of genes involved in adult OA resistance as predicted by Jones (2001).

### 4.2 Protein coding changes versus regulatory mutations affecting gene expression

The results of this study contribute to the growing body of work showing the importance of both regulatory mutations as well as protein coding mutations in the evolution of phenotypic traits. A mutation in the *cis*‐regulatory region of a gene allows for differences in gene expression while avoiding the negative effects of pleiotropy through differential expression among varying tissues, life stages, and environments. However, a protein coding change altering the function of that protein would affect an organism during all life stages. While an environment‐, stage‐, and tissue‐specific decrease in gene expression of *Osi6* and a stage‐specific increase of *Osi7* expression appear to contribute to only adult OA resistance, protein coding changes in *Osi8* may affect both larval and adult resistance. In our previous study, ubiquitous knockdown of *Osi6* and *Osi7* resulted in embryonic lethality, whereas knockdown of *Osi8* did not, consistent with only regulatory mutations affecting the expression of *Osi6* and *Osi7* and altered protein function of *Osi8 *(Andrade López et al., 2017). The modular nature of *cis*‐regulatory mutations (Gompel, Prud'homme B, Wittkopp PJ, Kassner VA, & Carroll SB, 2005) affecting *Osi6* and *Osi7* expression may allow for variable levels of gene expression in different life stages, while the protein coding changes in *Osi8* would potentially affect all life stages. The exact molecular and genetic basis of derived function and/or expression of *Osi6*, *Osi7* and *Osi8* remain unknown and require further investigation.

This protein CDS change fits in with other protein changes that appear to be involved in *D. sechellia's* host specialization, and is consistent with a proposed stepwise manner of evolution (Hungate et al., 2013). A protein coding change affecting host preference found in *D. sechellia* and functionally tested in *D. melanogaster* was investigated by Dworkin and Jones (2009) in which they discovered a premature stop codon resulting from a 7‐base deletion in the *D. sechellia* allele of *Obp56e*. *Drosophila melanogaster* showed reduced avoidance of *Morinda* fruit upon RNAi knockdown of *Obp56e* (Dworkin & Jones, 2009). Similarly, Matsuo, Sugaya, Yasukawa, Aigaki, and Fuyama (2007) found that the *D. sechellia* allele of *Obp57e* affected host preference due to a 4‐bp insertion upstream of this gene (Matsuo et al., 2007). If the loss of functional *Obps* was an initial step in the evolution of *D. sechellia's* host specialization on toxic *Morinda* fruit, a strong selection pressure to develop tolerance would exist on flies no longer avoiding and coming in contact with the fruit (Hungate et al., 2013). This is especially true for the larvae of *D. sechellia* as they would be chronically exposed to the toxic environment, having developed directly and exclusively feeding on the fruit. Another recent study suggested an evolved change in the catecholamine regulatory protein *Catsup* in *D. sechellia* along with the presence of L‐DOPA in *M. citrifolia* fruit has assisted the specialization of *D. sechellia* on its preferred host plant and found exposure to L‐DOPA altered resistance to Morinda fruit toxins, though the mechanism underlying this effect is still unknown (Lavista‐Llanos et al., 2014).

### 4.3 *Osi8* and a potential role in detoxification

Although little is known about the molecular function of most *Osiris* genes, sequence similarity between *Osiris* genes suggests they may serve similar functions. Lee et al. (2013) showed that loss of function of the *Osi21* protein shifts the membrane balance between late endosomes and lysosomes facilitating degradation of endocytosed rhodopsin in eye cells (Lee et al., 2013). This suppression of retinal degeneration in a *Drosophila* model of retinal dystrophy showed that loss of *Osi21* function negatively regulates endolysosomal trafficking. It is possible *Osi8* can serve a similar function as *Osi21* given they share conserved *Osiris* domains (Shah et al., 2012) including the duf1676 domain, a putative transmembrane domain which may localize the protein to the endomembrane system and potentially be involved in the dosage‐sensitive triple lethal locus (Dorer et al., 2003), along with a YXXø motif, a tyrosine‐based sorting signal that can mediate lysosomal targeting (Bonifacino & Dell'Angelica, 1999; Bonifacino & Traub, 2003). The loss‐of‐function mutations in *Osi21* include a single mutation (G149S) in the duf1676 domain. In our analyses, two mutations in the duf1676 domain (L95F & R129G) are derived in the *D. sechellia* allele of *Osi8* compared to susceptible sister taxa *D. simulans*. Furthermore, the sequence of *Osi8* is highly conserved with the L95 amino acid effectively unchanged across the entire *Drosophila* clade (~40 million years) and G149 amino acid unchanged across all insects (~300 million years) suggesting evolutionary constraint on these positions, yet *D. sechellia* has derived residues at both positions. We have shown that knockdown of *Osi8* expression results in increased susceptibility to OA in *D. melanogaster* larvae, matching previously published data showing the same RNAi result in adults (Andrade López et al., 2017). Together these data suggest *Osi8* and potentially *Osi6* and *Osi7* in adults may act as regulators of endolysosomal trafficking, contributing to the evolved OA resistance observed in *D. sechellia*. While *Osi8* resides within the fine‐mapped adult resistance region (Hungate et al., 2013) and potentially contributes to adult resistance (Andrade López et al., 2017), it does not fall directly beneath a QTL peak of major effect in larvae (Huang & Erezyilmaz, 2015). However, this QTL study investigated larval survival across multiple larval stages and found that the trait they were primarily observing and mapping was OA resistance during molting between larval instars. In our study, we focused on OA resistance in mid‐stage wandering L3 larvae, so it remains possible that *Osi8* could be involved in mid‐stage resistance despite there being no QTL peak over the *Osiris* locus for the mapped larval molt‐associated OA resistance. An additional QTL study will be needed focusing on the wandering L3 stage specifically to determine the contribution of the *Osiris* cluster to this trait.

Finding that knockdown of *Osi8* alters OA resistance in both *D. melanogaster* adults and larvae is an intriguing clue into *D. sechellia*'s host specialization and provides further support that members of the *Osiris* cluster contribute to OA resistance in *D. sechellia*. Additionally, confirming the derived mutations in *Osi8* are unique to *D. sechellia* and are not variable within wild‐caught strains of *D. sechellia* while being conserved in other insect species for over 300 million years of divergence time further suggests this gene is an evolved OA resistance factor. While testing gene function in model organisms like *D. melanogaster* is a commonly used practice, we are not directly testing gene function in *D. sechellia.* Therefore, we cannot rule out the possibility that these results will not perfectly translate among these closely related species. Further investigations focusing on the mechanism of *Osi8*‐mediated OA resistance using CRISPR/Cas9 gene editing are now needed. By swapping the *D. sechellia* *Osi8* protein coding sequence into *D. simulans* and the reciprocal swap of the *D. simulans* allele into *D. sechellia*, we will be able to confirm whether the protein coding changes in *Osi8* are responsible for the evolved OA resistance observed in *D. sechellia* across life stages. Further work is also needed to determine whether the biological mechanism of *Osiris* gene‐mediated toxin resistance acts through detoxification, bolstered physical barriers, another mechanism, or some combination of these factors.

## CONFLICT OF INTEREST

None declared.

## AUTHORS’ CONTRIBUTIONS

JDC and SML designed the experiments; RDP, SJS, and SML collected the data in the laboratory; and SCL and SML performed data analysis. JDC and SML designed figures and wrote the manuscript with feedback from RDP, SJS, and SCL.

## Data Availability

All octanoic acid resistance data and qRT‐PCR data generated in this study are archived at Dryad, and RNA‐seq data are archived at NCBI‐GEO (GSE123186).
