# Supplementary figures and images for: Investigating the role of Osiris genes in Drosophila sechellia larval resistance to a host plant toxin
Source: Ecol Evol. 2019 Jan 15;9(4):1922–33. doi: 10.1002/ece3.4885 (PMC6392368; doi:10.1002/ece3.4885)

Supplementary Figure 1

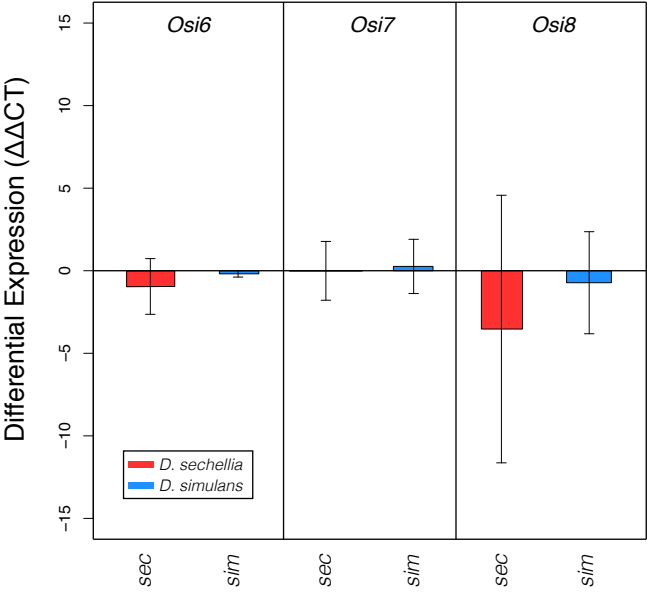

Supplement: Supplementary file 1 [file ECE3-9-1922-s001.pdf]

Supplementary Figure 2

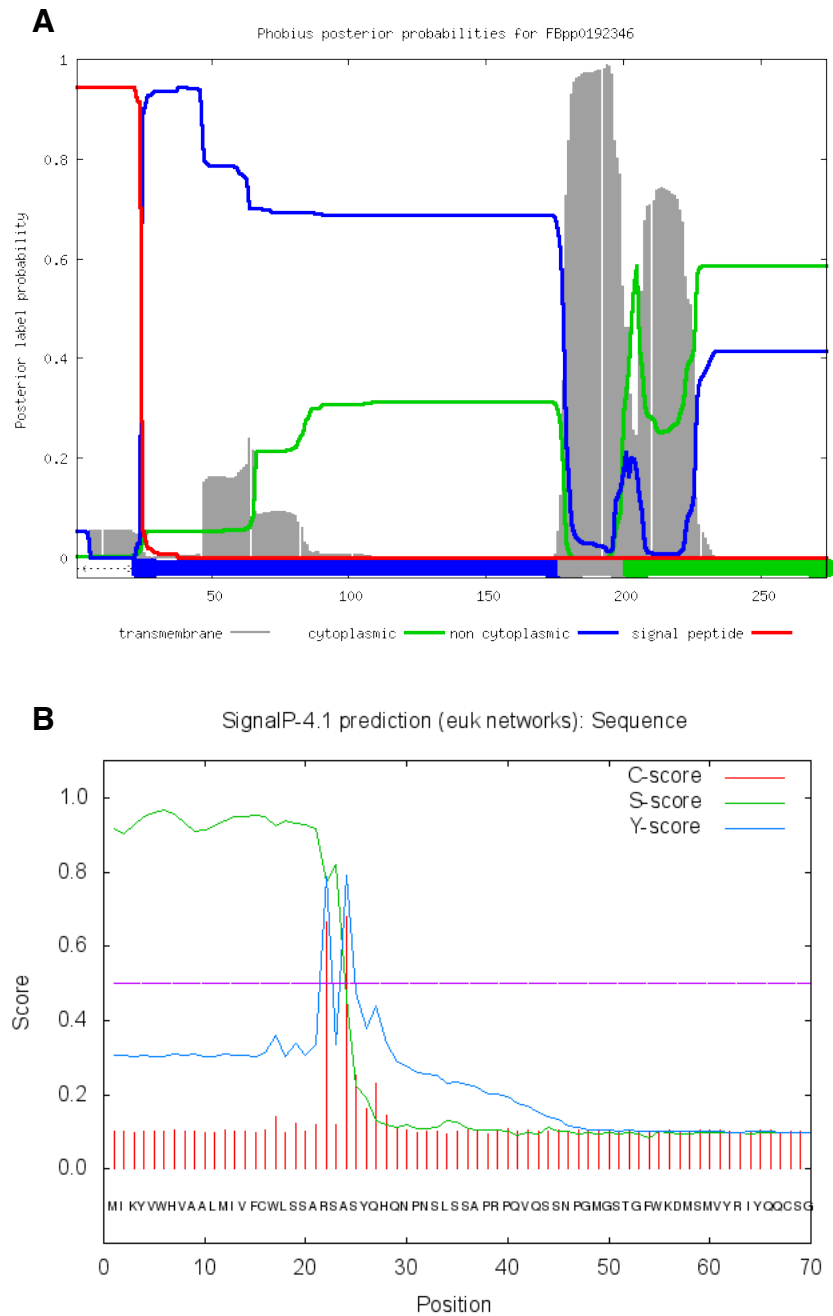

Supplement: Supplementary file 2 [file ECE3-9-1922-s002.pdf]
