# Supplementary material for: Investigating the role of Osiris genes in Drosophila sechellia larval resistance to a host plant toxin
Source: Ecol Evol. 2019 Jan 15;9(4):1922–33. doi: 10.1002/ece3.4885 (PMC6392368; doi:10.1002/ece3.4885)
Supplement: Supplementary file 3 [file ECE3-9-1922-s003.pdf]

Supplementary Figure 3

A

```
melanogaster      KLLTGLEKAFRSAKSLSLMEGIQFVSSG
suzukii           KLLTGLEKAFRSAKTLSLMEGIQFVSSG
simulans          KLLTGLEKAFRSAKSLSLMEGIQFVSSG
sechellia         KLLTGLEKAFRSAKSFSLMEGIQFVSSG
erecta            KLLTGLEKAFRSAKSLSLMEGIQFVSSG
yakuba            KLLTGLEKAFRSAKSLPLMEGIQFVSSG
ananassae         KLLTGLEKAFRSAKTLSLMDGIQFVSSG
pseudoobscura     KLLTGLEKVFRSAKTLALFEGVQFVSSG
persimilis        KLLTGLEKVFRSAKTLALFEGVQFVSSG
willistoni        KLLTGLEKAFRSAKSLTLFEGVQFVSSS
virilis           KLLTGLEKAFRTAKTLPLFEGIQFVGST
mojavensis        KLLTGLEKAFRSAKTLPLFEGIQFVSST
grimshawi         KLLTGLEKAFRTAKTFPLFEGIQFVSSG
                  ********.**:**:: *::*:***.*
```

B

```
melanogaster        ETKRAPISEKDIEAVLPRSVDAKEQVLN
suzukii             EAKRAPINEQDIEAVLPRSVDAKEQVLN
simulans            ETKRAPISEKDIEAVLPRSVDAKEQVLN
mauritiana          ETKRAPISEKDIEAVLPRSVDAKEQVLN
sechellia           ETKRAPISEKDIEAVLPGSVDAKEQVLN
erecta              EPKRAPVSEKDIEAVLPRGADAKEQVLN
yakuba              ETNRATVSEKDIEAVLPRSVDAKEQVLN
ananassae           EIKRAPISEQDIEAVLPRSIEAKDQVLN
pseudoobscura       RAKLPPISEQDIVAVLPRSTDAKDQVLN
persimilis          RAKLPPISEQDIVAVLPRSTDAKDQVLN
willistoni          RSKLPSISEQDIEAVLPRGVDAKEQVLN
virilis             RTQLPPITEQDIEAVLPRGLDAKEQILN
mojavensis          RSQLPPITEQDIEAVLPRGLDAKEQILN
grimshawi           RSQLPPISEQDIEAVLPRGLDAKEQILN
vitripennis         QPEEPIRSPQEIEASLPRSLEDKEDALN
aegypti             IDSTPVKSEAQIEMELPRSMDEKERSLN
gambiae             ADATPLKTEAQLEAELPRSLDEKERSLN
darlingi            LEVAPVKTEAQLELELPRSLDEKERSLN
quinquefasciatus    IDSTPVKSEAQLETELPRSMDEKERSLN
cuprina             LPTKPLLSEQELEANLPRALEAKEQVLT
morsitans           TKIKPILNEKDIEAVLPRALEAKEQTLN
plexippus           -------QKVEYEEAIPRGLDE--GSLD
castaneum           MTLNEVKTEEEYDATLPRALNERDTALN
                                 :    :*  . :     *
```
