# Supplementary material for: Investigating the role of Osiris genes in Drosophila sechellia larval resistance to a host plant toxin
Source: Ecol Evol. 2019 Jan 15;9(4):1922–33. doi: 10.1002/ece3.4885 (PMC6392368; doi:10.1002/ece3.4885)
Supplement: Supplementary file 4 [file ECE3-9-1922-s004.pdf]

Supplementary Figure 1: *Osiris 6*, *Osi7*, and *Osi8* are not significantly differentially expressed in response to 0.2% OA in *D. sechellia* and *D. simulans* larvae. Normalized relative gene expression levels of *Osi6*, *Osi7*, and *Osi8* in response to 0.2% OA measured in *D. sechellia* (red) and *D. simulans* (blue) larvae. Error bars represent 95% confidence intervals.

Supplementary Figure 2: *D. sechellia Osi8* signal and transmembrane predictions. (**A**) Signal peptide (red) and transmembrane (gray) prediction output by Phobius (v1.01). (**B**) Discriminating signal peptides (green, S-score) from transmembrane regions via SignalP (4.1).

Supplementary Figure 3: DNA CDS alignments of Osi8 with other Drosophila, non-Drosophila Dipterans, and non-Dipteran insects. (**A**) DNA coding sequence alignments of *Osiris 8* orthologs in other Drosophila species highlighting the L95F mutation observed in *D. sechellia*. (**B**) DNA coding sequence alignments of *Osiris 8* orthologs in other Drosophila species, non-Drosophila Dipterans, and non-Dipteran insects highlighting the unique R129G mutation observed in *D. sechellia*.

Table S1: Full genotypes of *Drosophila* lines used in this study.

Table S2: Gene-specific primer sequences.

Table S3: All significantly differentially expressed genes upon exposure to 0.2% OA.

Table S4: Significantly differentially expressed genes within larval OA resistance QTL peaks (Huang & Erezyilmaz 2015).

Table S5: Gene ontology term enrichment results for significantly differentially expressed genes in response to OA in *D. sechellia* larvae (.xlsx).

Table S6: SOSUIsignal output discriminating signal peptides from transmembrane helices.

Table S7: *Osiris 8* DNA variant calling summary of 23 wild-caught *D. sechellia* lines (.xlsx).

Table S1

**Gene**	**Stock**	**Genotype**	**Source**
Control	v60000	w^{1118}	VDRC
Osi5	v102392	P{KK110481}VIE-260B	VDRC
Osi6	v44545	w^{1118};P{GD2821}v44545	VDRC
Osi7	v8475	w^{1118};P{GD2822}v8475	VDRC
Osi8	v5753	w^{1118};P{GD2823}v5753	VDRC
edin	v109528	P{KK113306}VIE-260B	VDRC
LManVI	v15590	w^{1118}; P{GD5106}v15590	VDRC
C1GalTA	v110406	P{KK101417}VIE-260B	VDRC

Table S2

**Gene**	**Forward**	**Reverse**
αTub84B	TGTCGCGTGTGAAACACTTC	AGCAGGCGTTTCCAATCTG
Osi6	TCTTCCTGGCTCTGGCTGCT	TCTTGCCGCCGAAGAGACCT
Osi7	CGGCGGCGAGAACGACATTA	ACCGAGCACCTTGTCCACGA
Osi8	TGTCCGTTTGCCTAAAGGTCA	TCCTTGGCGTCCACACTTC

Table S3

***D. sechellia* gene**	**Control (FPKM)**	**OA (FPKM)**	**qvalue**	***D. melanogaster* ortholog**	**log2 C/B**	**chr**
GM12650	336.042	80.1379	0.0183569	*C1GalTA*	-2.068084949	2L
GM16820	421.308	126.625	0.0183569	*CG33128*	-1.734313042	2L
GM18483	43.4874	2.83554	0.0183569	*CG3355*	-3.938902043	2L
GM17342	43.5973	5.67119	0.0183569	*lectin-37Da*	-2.942515395	2L
GM12683	0.668244	6.73905	0.0183569	*LManVI*	3.334098346	2L
GM21465	57.1953	333.473	0.0183569	*AttC*	2.543601453	2R
GM21912	810.473	55.2887	0.0183569	*CG33998*	-3.87370757	2R
GM15545	130.407	13.7222	0.0183569	*CG9815*	-3.248437609	2R
GM21924	376.614	1340.79	0.0183569	*DptB*	1.831924758	2R
GM19911	186.255	583.999	0.0183569	*IM23*	1.648686744	2R
Mal-A1	0.846755	4.03422	0.045058	*Mal-A1*	2.252273255	2R

GM21609	765.169	3235.27	0.0183569	*Mtk*	2.080035787	2R
GM21673	30.4957	11.3796	0.0330425	*Tsf3*	-1.422155985	2R
GM13909	16.4596	57.5388	0.0330425	*CG10592*	1.805605857	3L
GM21621	39.1733	3.31311	0.0183569	*CG11353*	-3.563612659	3L
GM13850	3.51236	0.00001	0.0183569	*CG13299*	-18.4220812	3L
GM25578	382.442	17.8714	0.0183569	*CG42538*	-4.419516409	3L
GM14710	12.1848	44.8555	0.0330425	*CG5150*	1.880202322	3L
GM22250	24.7202	0.87915	0.0183569	*CG7365*	-4.813437267	3L
GM25706	84.2378	257.809	0.0183569	*edin*	1.613762965	3L
GM24474	3800.86	530.821	0.0183569	*Eig71Ea/d*	-2.840028534	3L
GM25550	5179.64	488.156	0.0183569	*Eig71Ea/d*	-3.407437661	3L
GM24473	4122.11	473.897	0.0183569	*Eig71Eb/c*	-3.120737571	3L
GM25549	4194.67	506.35	0.0183569	*Eig71Eb/c*	-3.05035046	3L
GM25551	1205.34	275.579	0.0183569	*Eig71Eg*	-2.128902297	3L
GM24471	1259.12	205.853	0.0183569	*Eig71Ej*	-2.612729406	3L
GM25416	17.9734	2.1714	0.0183569	*ImpL1*	-3.049165928	3L
GM26036	0.944161	5.11422	0.045058	*CG4830*	2.437409426	3R
GM26427	53.7206	13.0572	0.0183569	*CG7059*	-2.040629861	3R
GM24130	47.7998	19.3173	0.045058	*Cht5*	-1.307111121	3R
MtnB	7254.84	1996.22	0.0183569	*MtnC*	-1.861673071	3R
GM10611	485.074	136.977	0.0183569	*Spec2*	-1.824271184	3R
GM12900	218.495	52.2617	0.0183569	*Spn100A*	-2.063774306	3R
GM19212	1.37077	0.00001	0.0183569	*CG14052*	-17.064627	X
GM19130	67.2354	8.51907	0.0183569	*CG14770*	-2.980453174	X
GM12643	2595.84	488.654	0.0183569	*CG32762*	-2.409316256	X
GM12416	8445.83	1806.68	0.0183569	*CG4151*	-2.22489821	X
GM13371	24.4769	73.2093	0.045058	*CG9672*	1.580606078	X
GM19198	14.6	4.9869	0.0183569	*csw*	-1.54975319	X

Table S4

D. sechellia gene	*D. melanogaster* ortholog	Expression in C (FPKM)	Expression in OA (FPKM)	Larval QTL
GM25578	*CG42538*	382.442	17.8714	QTL-III$_{sec}$a
GM22250	*CG7365*	24.7202	0.87915	QTL-III$_{sec}$a
GM25706	*edin*	84.2378	257.809	QTL-III$_{sec}$a
GM24474	*Eig71Ea/Eig71Ed*	3800.86	530.821	QTL-III$_{sec}$a
GM25550	*Eig71Ea/Eig71Ed*	5179.64	488.156	QTL-III$_{sec}$a
GM24473	*Eig71Eb/Eig71Ec*	4122.11	473.897	QTL-III$_{sec}$a
GM25549	*Eig71Eb/Eig71Ec*	4194.67	506.35	QTL-III$_{sec}$a
GM25551	*Eig71Eg*	1205.34	275.579	QTL-III$_{sec}$a
GM24471	*Eig71Ej*	1259.12	205.853	QTL-III$_{sec}$a
GM12643	*CG32762*	2595.84	488.654	QTL-X$_{sec}$
GM12416	*CG4151*	8445.83	1806.68	QTL-X$_{sec}$
GM12650	*C1GalTA*	336.042	80.1379	QTL-II$_{sim}$a
GM12683	*LManVI*	0.668244	6.73905	QTL-II$_{sim}$a
GM15545	*CG9815*	130.407	13.7222	QTL-II$_{sim}$b
GM13909	*CG10592*	16.4596	57.5388	QTL-III$_{sim}$b
GM21621	*CG11353*	39.1733	3.31311	QTL-III$_{sim}$b
GM14710	*CG5150*	12.1848	44.8555	QTL-III$_{sim}$b

Table S6

No.	N terminal	transmembrane region	C terminal	type	length
1	1	MIKYVWHVAALMIVFCWLSSA	21	SignalPeptide(PRIMARY)	21
2	180	MIMIPLLLGGTIVPLAYGALAML	202	PRIMARY	23
3	210	SKLALVLASIIGIKKLLSGGGGG	232	SECONDARY	23
